# 2192. Impact of an Antibiotic Transitions of Care Program Utilizing Outpatient Retail Pharmacists

**DOI:** 10.1093/ofid/ofad500.1814

**Published:** 2023-11-27

**Authors:** Jeannette Bouchard, Alison Orvin, Adam Cochran, Kelly Taylor, Renee Eldridge, Azima Habibfeisal

**Affiliations:** WakeMed Health and Hospitals, Raleigh, North Carolina; WakeMed Health and Hospitals, Raleigh, North Carolina; WakeMed Health and Hospitals, Raleigh, North Carolina; WakeMed Health and Hospitals, Raleigh, North Carolina; WakeMed Health and Hospitals, Raleigh, North Carolina; WakeMed Health and Hospitals, Raleigh, North Carolina

## Abstract

**Background:**

For many common community infections shorter durations of therapy are equivalent to longer durations and can be completed in 7 days or less. There’s a growing need to target antibiotic prescribing at discharge and reduce total length of antibiotic therapy. The objective of this study was to determine the impact of a transitions of care (TOC) program with outpatient retail pharmacists.

**Methods:**

This retrospective cohort study included patients ≥18 years discharged with antibiotic prescriptions to the hospital’s outpatient pharmacy from August 2021 to June 2022. Prescriptions included had to be for the following infections: community acquired pneumonia (CAP), acute COPD exacerbation, uncomplicated urinary tract infections, complicated urinary tract infections, pyelonephritis, and cellulitis. Patients were excluded if they were immunocompromised, had an Infectious Diseases consult, a Urology consult, or had bacteremia at any point during admission. The intervention group contained prescriptions reviewed by outpatient pharmacists utilizing a protocol, the control group prescriptions were unreviewed. The primary outcome was adherence to protocol durations of therapy. Secondary outcomes include average outpatient prescription duration, number of recommendations made, and number of recommendations accepted.

**Results:**

323 patients were screened for inclusion, 162 in the intervention group and 160 in the control group. Seventy-nine (49%) and 76 (48%) were included in the intervention group and control groups, respectively. Overall, 59% were female, mean age was 67, most frequent indications were CAP (36%) and cellulitis (22%). Adherence to protocol durations were 75% and 50% in the intervention and control groups, respectively (p< 0.01). The average outpatient prescription duration was 4 days for the intervention group and 4.5 days for the control group. 40 (51%) of prescriptions in the intervention group had recommendations made and 28 (70%) were accepted. Most recommendations were for CAP (38%).

Average Total Antibiotic Duration and Maximum Allowed Duration Per Protocol

**Figure:**
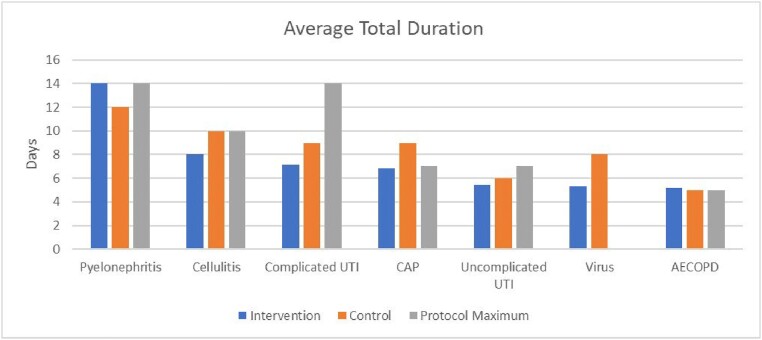

**Conclusion:**

The TOC program with outpatient pharmacists had better adherence to protocol durations of therapy than control and acceptance rates were high among recommendations made. This program showcases a potential avenue to improve outpatient antibiotic durations.

**Disclosures:**

**All Authors**: No reported disclosures

